# Management of spinal deformities and tibial pseudarthrosis in children with neurofibromatosis type 1 (NF-1)

**DOI:** 10.1007/s00381-020-04775-4

**Published:** 2020-07-01

**Authors:** Kiril V. Mladenov, Alexander Simon Spiro, Kara Leigh Krajewski, Ralf Stücker, Philip Kunkel

**Affiliations:** Altona Children’s Hospital – AKK/UKE, Bleickenallee 38, 22763 Hamburg, Germany

**Keywords:** Neurofibromatosis type 1, Dystrophic scoliosis, Congenital pseudarthrosis of the tibia

## Abstract

**Summary of background data:**

The skeletal system is affected in up to 60% of patients with neurofibromatosis type 1. The most commonly observed entities are spinal deformities and tibial dysplasia. Early recognition of radiologic osseous dystrophy signs is of utmost importance because worsening of the deformities without treatment is commonly observed and surgical intervention is often necessary. Due to the relative rarity and the heterogenic presentation of the disease, evidence regarding the best surgical strategy is still lacking.

**Purpose:**

To report our experience with the treatment of skeletal manifestations in pediatric patients with (neurofibromatosis type 1) NF-1 and to present the results with our treatment protocols.

**Materials and methods:**

This is a retrospective, single expert center study on children with spinal deformities and tibial dysplasia associated with NF-1 treated between 2006 and 2020 in a tertiary referral institution.

**Results:**

*Spinal deformity:* Thirty-three patients (*n* = 33) were included. Mean age at index surgery was 9.8 years. In 30 patients (91%), the deformity was localized in the thoracic and/or lumbar spine, and in 3 patients (9%), there was isolated involvement of the cervical spine. Eleven patients (33%) received definitive spinal fusion as an index procedure and 22 (67%) were treated by means of “growth-preserving” spinal surgery. Halo-gravity traction before index surgery was applied in 11 patients (33%). Progression of deformity was stopped in all patients and a mean curve correction of 60% (range 23–98%) was achieved. Mechanical problems with instrumentation requiring revision surgery were observed in 55% of the patients treated by growth-preserving techniques and in none of the patients treated by definitive fusion. One patient (3%) developed a late incomplete paraplegia due to a progressive kyphotic deformity.

*Tibial dysplasia:* The study group comprised of 14 patients. In 5 of them (36%) pathological fractures were present on initial presentation. In the remaining 9 patients (64%), anterior tibial bowing without fracture was observed initially. Four of them (*n* = 4, 28%) subsequently developed a pathologic fracture despite brace treatment. Surgical treatment was indicated in 89% of the children with pathological fractures. This involved resection of the pseudarthrosis, autologous bone grafting, and intramedullary nailing combined with external fixation in some of the cases. In 50% of the patients, bone morphogenic protein was used “off-label” in order to promote union. Healing of the pseudarthrosis was achieved in all of the cases and occurred between 5 to 13 months after the index surgical intervention. Four of the patients treated surgically needed more than one surgical intervention in order to achieve union; one patient had a re-fracture. All patients had a good functional result at last follow-up.

**Conclusion:**

Early surgical intervention is recommended for the treatment dystrophic spinal deformity in children with NF-1. Good and sustainable curve correction without relevant thoracic growth inhibition can be achieved with growth-preserving techniques alone or in combination with short spinal fusion at the apex of the curve. Preoperative halo-gravity traction is a safe and very effective tool for the correction of severe and rigid deformity in order to avoid neurologic injury. Fracture union in tibial dysplasia with satisfactory functional results can be obtained in over 80% of the children by means of surgical resection of the pseudarthrosis, intramedullary nailing, and bone grafting. Wearing a brace until skeletal maturity is achieved is mandatory in order to minimize the risk of re-fracture.

## Spinal deformity

### Introduction

Spinal deformity (scoliosis, kyphosis) is the most common skeletal manifestation in patients with neurofibrosis type 1 (NF-1) and has been described in up to 60% of individuals affected [1]. The pathophysiological mechanism of bone involvement is still unknown; however, intraosseous neurofibromas, mesodermal dysplasia, osteomalacia, or endocrine factors have all been hypothesized to play a role [2].

Depending on the morphological aspects of the osseous changes, it is of utmost importance to differentiate between “non-dystrophic” and “dystrophic” curves. The early recognition of dystrophic features is mandatory because these features can be used to predict the risk of deformity progression and play a significant role in the establishment of the treatment strategy [3].

The typical dystrophic changes are summarized in Table 1 and in Figs. 10 and 11.

**Table 1:** Radiographic features of dystrophic changes in NF-1

Feature	Location	Description
Scalloping	Vertebral body (posterior, anterior or lateral)	Depth of scalloping > 3 mm thoracic spine > 4 mm lumbar spine
Rotation	Vertebra	
Penciling	Rib	Width of the rib is smaller than that of the narrowest portion of the second rib
Wedging	Vertebral body	Similar to congenital hemivertebra
Spindling	Transverse process	TP thinned like a spindle
Widening interpedicular distance	Spinal canal	Seen in the AP projection compared to the adjacent vertebra
Enlargement	Neuroforamen	Seen in the lateral view compared to the adjacent vertebra
Paravertebral mass		Seen mostly on MRI

Since the disease is rare and because spinal deformities can have very heterogenic morphological patterns, current treatment recommendations are based on experience from the past. However, well-established treatment protocols based on long-term data and large patient cohorts are still lacking and currently there is no evidence about the best treatment modality. Prospective, randomized, controlled studies are needed to define study protocols; however, the performance of such studies is extremely difficult due to the heterogeneity of presentation, small patient cohorts, and ethical issues. The availability of RCT data is not to be expected in the near future. The current manuscript presents the results and describes our recommendations for the treatment of spinal deformities in pediatric patients with NF-1 based on our long-term experience in the management of these patients.

### Materials and methods

We performed a retrospective review of the clinical records, radiographs, and other imaging studies of all patients treated at our institution between 2006 and 2020 for spinal deformities associated with NF-1.

Patient demographics were gathered from the charts and clinical records were studied for type and number of surgical procedures as well as for intra-, perioperative and late complications.

The location and type of the deformities as well as the presence of dystrophic changes were studied on initial radiographs. The extent of coronal and sagittal spinal involvement was evaluated by means of standardized measurement of the main curve according to the Cobb method on upright radiographs performed before surgery (before application of halo-gravity traction in applicable cases), immediately postoperatively and at latest follow-up. Fusion was analyzed on the latest imaging study (X-ray, MRI, CT). In the group treated with growth-preserving techniques, the height of T1–T12 was measured on postoperative and most recent AP radiographs in order to evaluate spinal growth. Patients were divided into groups according to the location of the deformity (cervical vs. thoracic and/or lumbar), type of procedure (growth preserving vs. definitive fusion), and type of surgical approach (anterior, posterior, or combined).

### Results

Thirty-three patients fulfilled the inclusion criteria (*n* = 33). Mean age at index surgery was 9.8 years (range 4.3–16.6 years). Thirty patients (*n* = 30) had a spinal deformity located in the thoracic and/or lumbar spine. In the remaining three patients (*n* = 3), the deformity was located in the cervical spine. Definitive fusion was performed at the time of the index procedure in 11 patients: anterior spinal fusion (ASF) *n* = 1, posterior spinal fusion (PSF) *n* = 7, combined anterior and posterior spinal fusion (APSF) *n* = 3. A growth-preserving technique (GPT) as a stand-alone procedure was performed as the initial procedure in 11 patients: magnetic controlled growing rods (MCGR) *n* = 5, vertical expansion prosthetic titanium ribs (VEPTR) *n* = 6. Seven patients (*n* = 7) received a combination of a short fusion and a growth-preserving procedure (in the same anesthesia setting—3 cases, staged or pending in 3 cases). Five patients initially treated by growth-preserving procedures were converted into definitive fusion after skeletal maturity. All 3 patients with cervical deformities presented with kyphosis and were treated by means of APSF. Eleven patients had preoperative halo-gravity traction (HGT). In 8 patients recombinant human bone morphogenic protein type 2 (rhBMP-2) was used (at the time of the index procedure *n* = 3, and at the time of revision or last procedure *n* = 4). The major scoliosis curve measured prior to surgery had a mean of 70° (range 51–96°) and was corrected to 28.6° (range 1–55°) at the latest follow-up, resulting in a correction rate of 60% (range 23–98%). Mean cervical kyphosis measured 97° (range 70–125°) initially and was corrected to 25° (range 10–52°) at latest follow-up, representing a mean correction of 77% (range 59–87%). In all cases, the curve was corrected and progression of deformity could be stopped. Patients treated with GPT showed an average longitudinal growth of the thoracic spine of 7.65 mm/year.

Mechanical complications occurred in 12 patients treated with GPT and were mostly due to aseptical loosening of the rib anchors. In these cases, revision surgery was necessary. Five of the patients needed more than one revision surgery for mechanical issues. A total of two patients (*n* = 2) developed neurological symptoms. In one of them, these were not related to the treatment of the spinal deformity and were due to peripheral neuropathy caused by systemic anti-tumor therapy for malignant transformation of neurofibromas. In the second patient, incomplete lower paraplegia developed 10 years after primary surgery for deformity correction. This was due to progressive dystrophic bone changes representing the characteristic “NF-1-related modulation process,” which caused an increasing kyphotic deformity. The deformity was treated by removal of the instrumentation and the application of HGT for a slow correction of kyphosis over 4 weeks followed by anterior and posterior spinal fusion and anterior strut grafting in the same surgical setting. Partial recovery of the paraplegia was observed 6 months after surgery. At latest follow-up, the patient was able to ambulate short distances with a posterior walker. Patient data are summarized in Table 2.

**Table 2:** Summary of patient data on spinal deformities associated with NF-1

No.	Age initial (years)	Follow-up years	Diagnosis	Initial surgery	Following surgery	Pre-op HGT	BMP	No. of revision surgeries	Curve initial	Curve last	% corr	T1–T12 (mm/year)
1	5.5	3.5	Scoliosis	APSF T2–T9	MCGR	+			88	43	52	8
2	12.8	4.5	Scoliosis	PSF T2–L1					78°	23	71	
3	11.2	7	Scoliosis	VEPTR (2013)	PSF T2–L3 (2018)				79	28	65	10
4	7.3	7	Scoliosis	MCGR (2013)	PSF (2020)		last	1 × MCGR	64	43	23	5.9
5	12.1	3	Scoliosis	PSF T2-9 (2017)					51	8	85	
6	6.6	6	Kyphosis/scoliosis	MCGR (2014)	APSF, Strut (2018)	+		1 × MCGR	73	35	53	
7	8.3	8	Scoliosis	MCGR (2012)				1 × MCGR	50	29	42	8.1
8	7.7	8	Scoliosis	MCGR (2012)				1 × MCGR exchange	64	41	36	6.2
9	8	6.5	Scoliosis	MCGR (2013)	PSF T2–L3 (2019)			1 × Anchor, 1 × MCGR	51	25	51	9.1
10	16	2	Scoliosis	PSF (T6–L3) (2017)					68	28	59	
11	11.1	4.5	Scoliosis	VEPTR				3	71	22	70	12
12	10.5	6	Scoliosis	VEPTR/PSF(T12–L2) (2011)	PSF T1–L2 (2019)			2 × VEPTR	72	55	24	7.4
13	4.7	3	Kyphosis/scoliosis	ASF (T10–T11) MCGR		+	Index	2 × Rib Anchor	81	18	78	7.3
14	12.1	4	Scoliosis	VEPTR/PSF (T12–L3) (2015)	PSF T2–L4 (2018)			1 × Rib Anchor	84	39	54	7
15	9.1	2	Kyphosis C-spine	APSF (C2–C6		+						
16	4.7	3.5	Scoliosis	APSF (T11–L4) VEPTR		+			73	19	74	6.9
17	13.1	2	Scoliosis	APSF (T1–12)		+			96	12	88	
18	4.9	12	Scoliosis	VEPTR	PSF, APSF, Strut	+	Rev	4x	88	46	48	7.1
19	15.1	2	Kyphosis C-spine	APSF (C3–C7)		+						
20	11.5	1.5	Scoliosis	APSF (T12–pelvis)			Index		67	8	89	
21	5.8	2	Scoliosis	ASF (T7–T9)	MCGR pending				57	27	53	
22	11.9	1	Scoliosis	PSF (T10–L4)					49	1	98	
23	10.2	1	Scoliosis	ASF (T8–T11)	MCGR				79	44	55	
24	8.1	1	Scoliosis	ASF(T5–T7)	MCGR pending				56	24	58	
25	16.6	1	Kyphosis/scoliosis	APSF(C2–T12), Strut(T2–T6)		+	Index/rev					
26	4.3	9	Scoliosis	VEPTR								
26a	4.3	9	Kyphosis C-spine	ASF Cervical	APSF Cervical	+	Rev		87	35	60	5.1
27	9.8	7.5	Scoliosis	VEPTR	PSF T2–L4		last		71	25	65	8.5
28	14	3	Scoliosis	PSF (2012)					58	16	73	
29	10.2	2	Scoliosis	APSF					78	27	66	
30	5.9	9	Scoliosis	VEPTR (2006)	PSF T2–L3 (2013)	+	last	1 × VEPTR	73	38	48	7.8
31	7.5	6.5	Scoliosis	VEPTR				1 × VEPTR	81	51	38	6
32	14.1	4.5	Scoliosis	PSF (T7–L3)					59	22	63	7.65
33	12.8	4.5	Scoliosis	PSF (T3–T8) 2013	PSF T6–L2				62	26	59	

### Discussion

Since the characteristics of the deformities affecting different spinal regions (cervical spine vs. thoracic and lumbar spine) differ significantly from each other in terms of specific issues and treatment strategies, these entities will be discussed separately.

#### Cervical spine

The cervical spine in NF-1 patient needs particular attention, since involvement has been reported in up to 30% of the patients presenting with spinal deformity [4]. A co-existing cervical entity can easily be overlooked since initial attention is paid to the more striking thoracic or lumbar deformities. Moreover, patients with cervical deformities are usually asymptomatic. The first presenting clinical symptom is intermittent neck pain and the most common deformity is cervical kyphosis. A thorough evaluation of the C-spine is mandatory in every patient presenting with NF-1 and includes questioning about complaints, clinical evaluation for tumor mass or incisional scars from past surgical interventions, clinical evaluation for the presence of deformities, evaluation of range of motion as well as a full neurological exam. AP and lateral X-rays should be obtained if one of the abovementioned criteria is not within normal limits. Furthermore, imaging of the C-spine is recommended before endotracheal intubation or a HGT in order to exclude potential instability and to prevent inadvertent spinal cord damage. It should be emphasized that standard flexion-extension X-rays are insufficient for the exclusion of instability since their interpretation is difficult due to the complexity of the deformity and the presence of dystrophic changes. For this purpose, a thin-slice CT scan with sagittal and coronal reconstructions and/or a flexion-extension MRI is necessary and should be performed. Significant or progressive C-spine deformity as well as underlying or impending instability in a patient with NF-1 is treated surgically. Different surgical approaches have been discussed in the literature comprising anterior only, posterior only or combined fusion [5]. In our experience, a combined anterior and posterior fusion with instrumentation is the method of choice. “In situ” fusion alone is performed only in the very rare case of little or no deformity. All patients in our series had dystrophic curves with significant kyphosis exceeding 45°. In these cases, preoperative halo-gravity traction for 4 weeks followed by combined anterior and posterior fusion in the same surgical setting was performed.

**Fig. 1:**
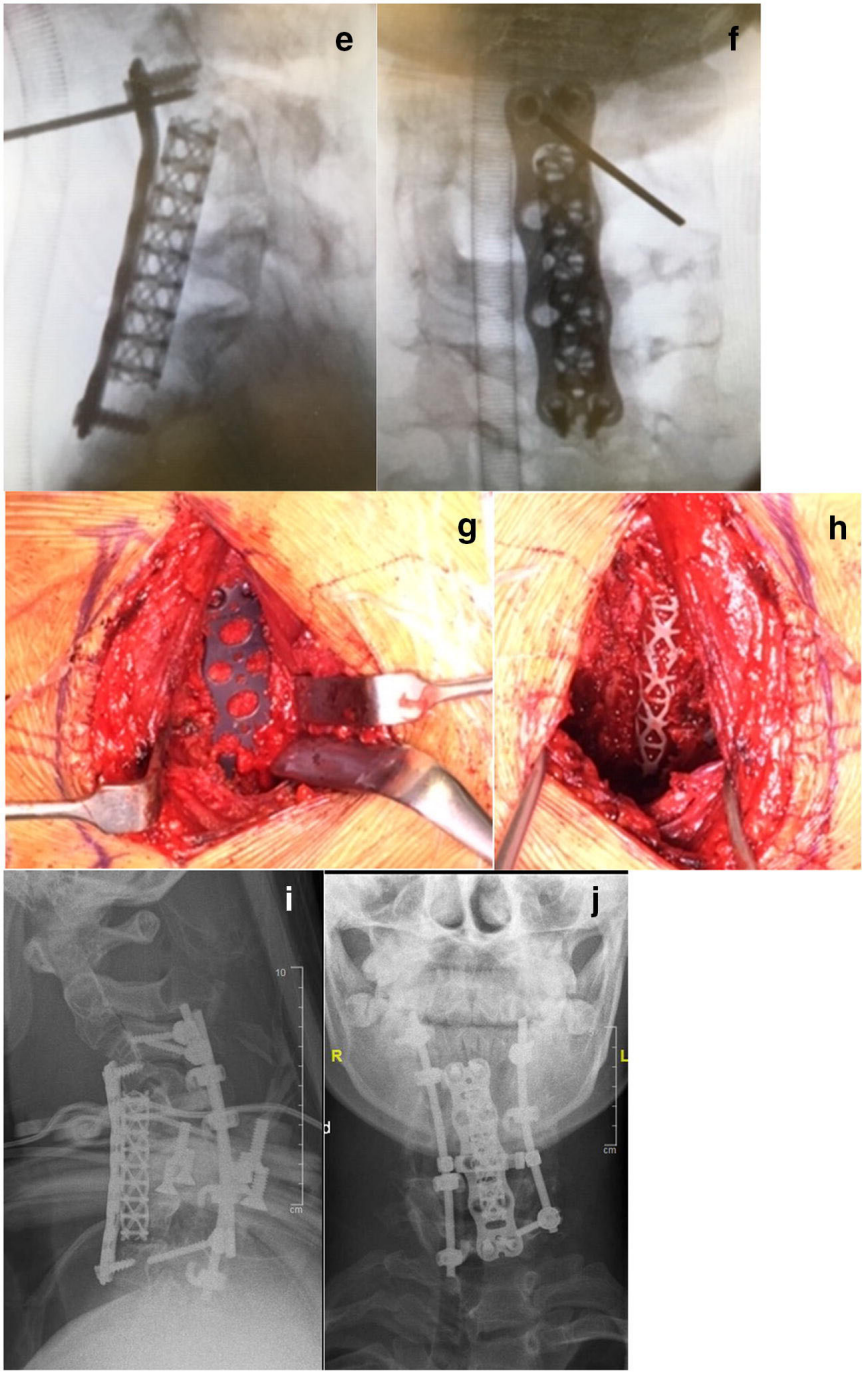

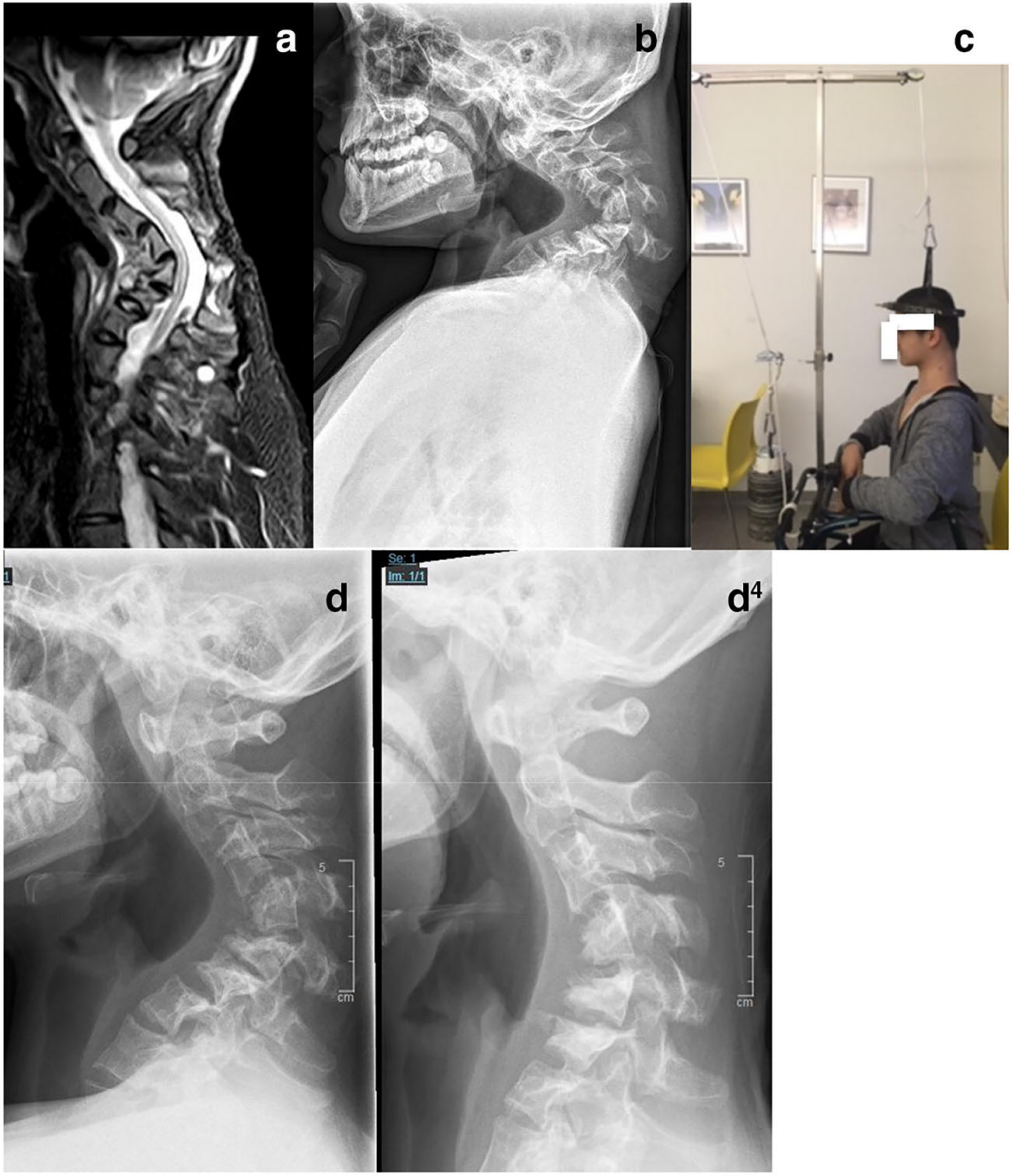
(Case 1) Lateral MRI (**a**) and plain X-ray films (**b**) of a 15-year-old patient with NF-1 showing severe kyphosis of the C-spine. Clinical image in preoperative halo-gravity traction (**c**). X-rays 2 weeks and 4 weeks (**d**) after the beginning of the HGT. Intraoperative image intensifier images after anterior cage and plate insertion (**e**, **f**) and clinical photos of the surgical site (**g**, **h**). Postoperative X-rays in a halo jacket (**i**, **j**)

Meticulous removal of the intervertebral discs and generous autologous cancellous bone application is of utmost importance in order to achieve union. If the height of the anterior column is preserved, then an anterior screw plate can be used. In cases with significant dystrophic alterations of the vertebral bodies, a structural anterior support is necessary in order to restore height of the anterior column. In all our cases, a mesh titanium cage filled with autologous cancellous bone was used. Additionally, posterior fusion with instrumentation was performed. Depending on the morphology and the quality of the posterior vertebral structures a double rod construct combined with lateral mass screws, laminar hooks or sublaminar bands can be used (Fig. 1).

Autologous cancellous bone from the iliac crest was applied and all patients were immobilized after surgery in a halo vest until solid union was confirmed by means of a CT scan. Two patients showed solid union after 3 months. The remaining patient developed a delayed union. An augmentation procedure including application of rhBMP-2 was performed. Solid union was observed 8 weeks after augmentation surgery.

#### Thoracic and lumbar spine

The principles of treatment for thoracic and lumbar deformities do not differ substantially from each other.

**Fig. 2:**
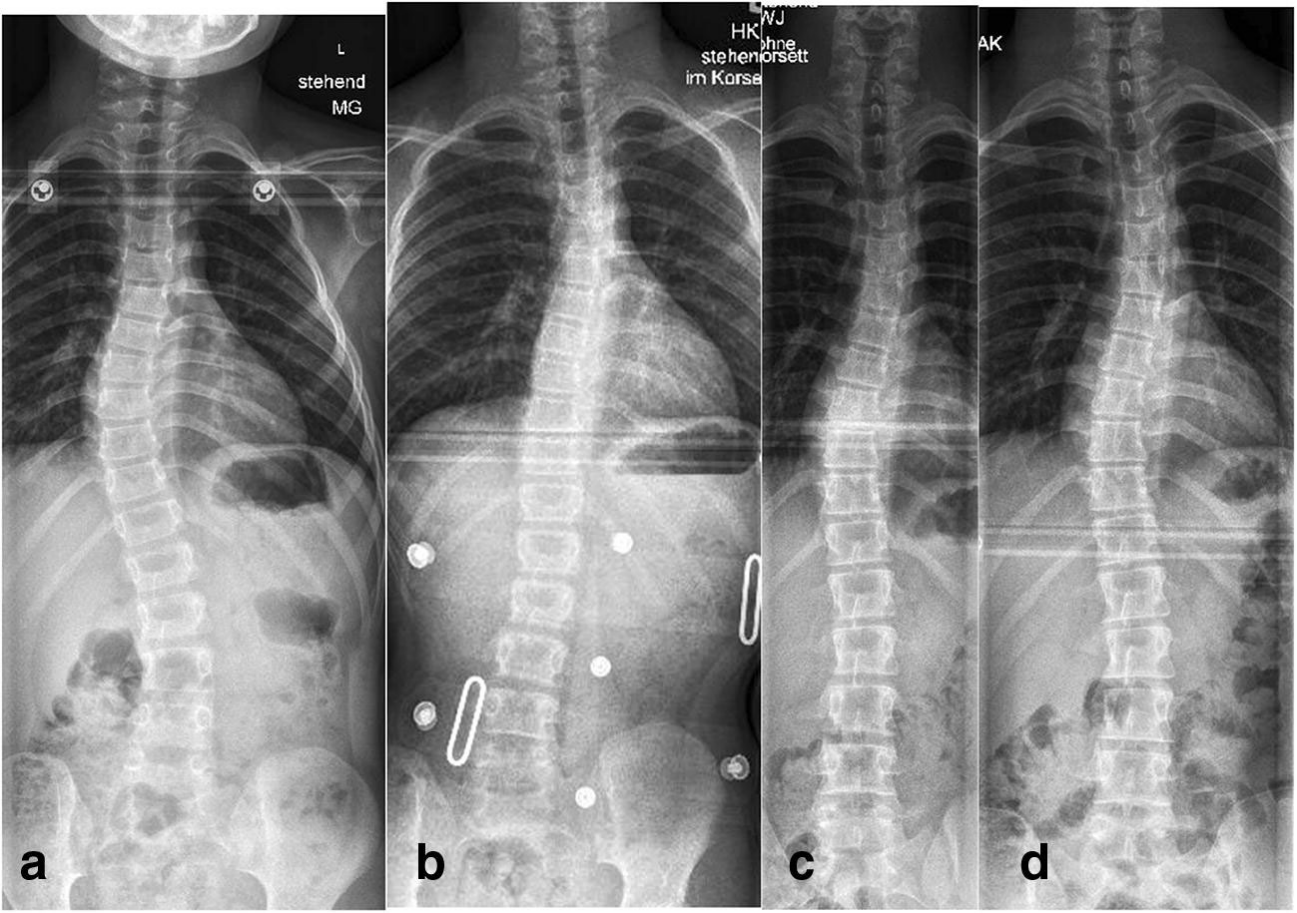
(Case 2) Eight-year-old patient with an “idiopathic-like” early-onset scoliosis (**a**–**d**). The curve showed mild progression to 27° (**a**). Brace treatment was initiated and good curve correction could be achieved (**b**). Full-time brace wear was recommended and further worsening of the scoliosis was prevented until skeletal maturity was reached. The brace treatment was discontinued at age 15 (**c**). One year after brace discontinuation: the curve remained unchanged (**d**)

Non-dystrophic NF-1 scoliotic curves are also called “idiopathic-like” because of their non-progressive or slowly progressive “benign” course. Treatment protocols are similar to idiopathic scoliosis. A non-progressive curve of up to 25° without dystrophic features in an immature patient is simply clinically and radiologically observed every 6 months. If progression occurs, a full-time Cheneaux-type brace is prescribed and used until skeletal maturity is reached (Fig. 2).

It should be emphasized that every subsequent X-ray must be thoroughly analyzed for the appearance of novel dystrophic changes since these may develop with time in an initially non-dystrophic case. This process is known as “modulation” and is an important negative prognostic factor for curve progression [6].

As opposed to non-dystrophic curves, the natural history of dystrophic spinal deformity in NF-1 patients is one of inevitable deterioration, especially if left untreated [7, 8].

Brace treatment of a dystrophic deformity is always unsuccessful and it has been stated that “There is no justification to observe the dystrophic curve in NF-1 because it always progresses” [9, 10].

The purpose of surgical treatment is to obtain a well-balanced, stable, functional spine and to preserve neurological function.

Surgical strategy depends on “curve-specific” and “patient-specific” factors.

Among the curve-specific factors are the severity and the rigidity of the deformity, the extent of dystrophic bone alterations, and the presence of paraspinal or intraspinal abnormalities such as tumors, dural ectasia, meningocele, etc. Patient-specific factors include expected residual growth, general condition, nutritional status, co-morbidities, accompanying malignant transformation of neurofibromas, etc.

Spinal fusion is the best option to correct the deformity, to stop progression, and to achieve stability in a dystrophic curve. However, there is evidence that spinal fusion in an immature individual with early-onset scoliosis (EOS) carries a risk of spinal and lung growth inhibition resulting in a respiratory restrictive disease known as “thoracic insufficiency syndrome” (TIS) [11].

It has been reported that the negative effects of early spine fusion on thoracic growth is directly related to the number of the levels fused [12].

Thus, in a patient younger than 10 years, fusion should be avoided or performed on as few segments as possible in order to preserve thoracic and pulmonary growth. However, there is still no consensus about the type of procedure in patients with EOS and NF-1. Some authors have recommended growing rods as a stand-alone strategy and reported curve corrections of 51% and successful preservation of spinal growth (T1–S1) with a mean of 11.2 mm/year (normal 12–17 mm). However, the reported rate of implant-related complications was relatively high (57%), though not to a higher extent than in other conditions causing EOS. Mechanical failure of proximal anchors was found to be the most common complication occurring in 35% of the cases [13].

In a most recent study, good long-term results after circumferential anterior and posterior spinal fusion (APSF) in 11 immature patients with NF-1 who had surgery at an average age of 8.4 years was reported. Curve correction averaged 67% and remained unchanged at latest follow-up of 14 years. Despite the progression of dystrophic changes, solid fusion was documented in all cases. Lung function remained almost unchanged (75.0 vs. 74.4% of predicted). However, growth inhibition was significant and spinal growth (T1-S1) was less than expected and averaged only 3.9 mm per year [14]. It is obvious that compared with definitive fusion, growing rod surgery preserves growth, but it is associated with a higher incidence of implant-related complications (IRCs) and lower correction rates for scoliosis associated with NF-1. Our approach to a dystrophic curve in an immature patient is to perform a short anterior (preferred) or posterior fusion comprising only the dystrophic levels. In our opinion, a fusion of up to 5 levels does not lead to significant growth disturbance since normal growth in the dystrophic segments cannot be expected. Insufficient curve correction or curve deterioration would diminish thoracic height even more. Furthermore, deformity correction aims not only at straightening of the spine but concurrently at restoration of thorax symmetry, which is important for the normal mobility of the chest wall during breathing excursions.

**Fig. 3:**
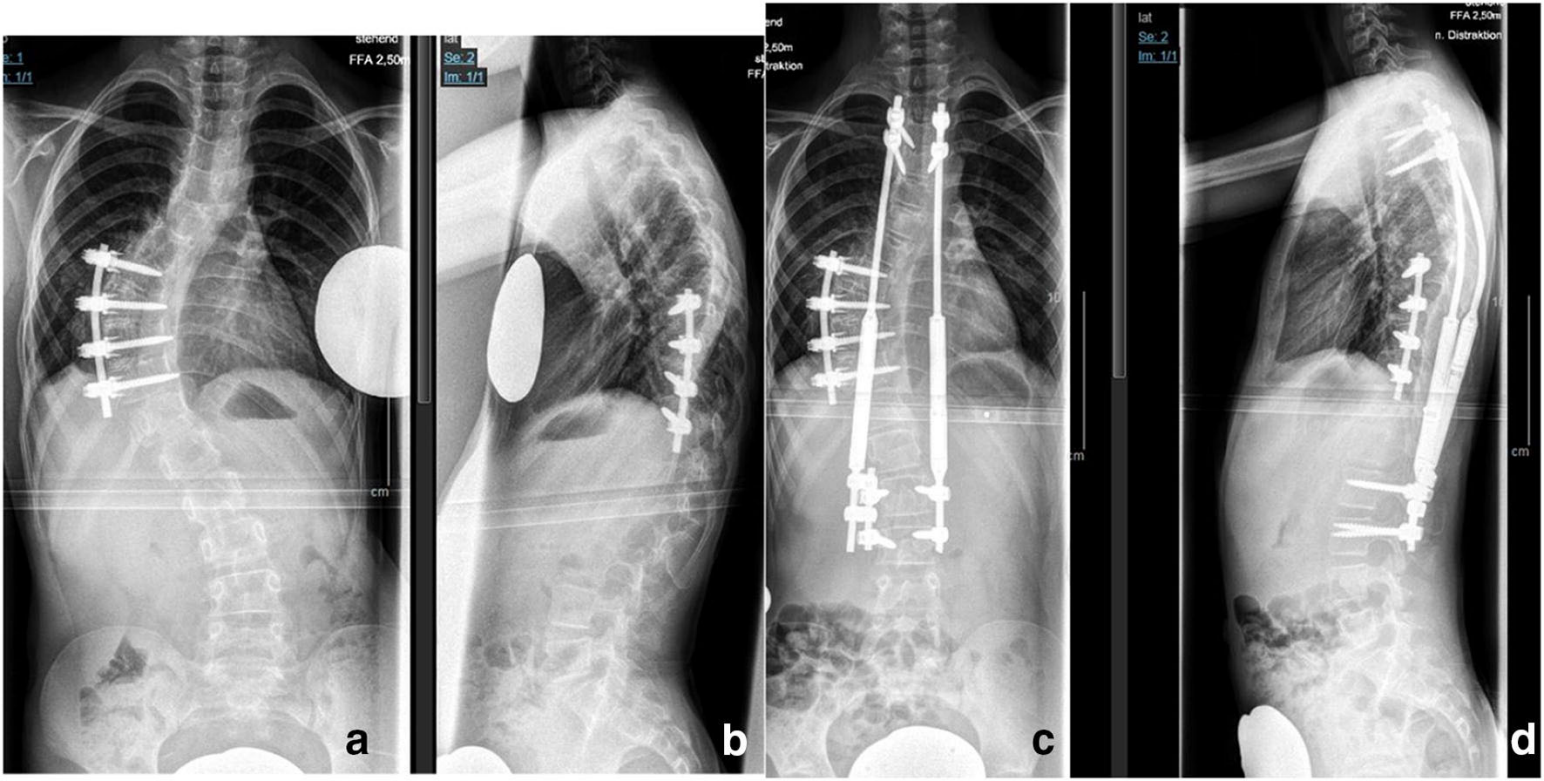
(Case 3) A nine-year-old NF-1 patient with progressive dystrophic scoliosis (**a**, **b**). A short segment anterior apical fusion (T8–11) was performed to correct apical deformity and achieve stable union (**a**). In a second surgery, growth-preserving, vertebra-based MCGR instrumentation (T3–L3) was used in order to correct residual long segment scoliosis and global kyphosis (**b**)

Our results with this approach are very good. In our series, the mean curve before surgery measured 72° and was corrected to 33° at latest follow-up, representing a curve correction of 54%. Growth of the thoracic spine (T1–12) was preserved to an almost normal average rate of 7.65 mm/year (normal range 7–11 mm) (Fig. 3).

The current surgical strategies after skeletal maturity are based on deformity correction and stable union through an anterior, posterior or combined approach.

A sole posterior approach is generally recommended for mild curves < 60°. Even in curves greater than 60°, a posterior approach may be a good option provided that the deformity is very flexible and straightens to < 25° on side bending.

**Fig. 4:**
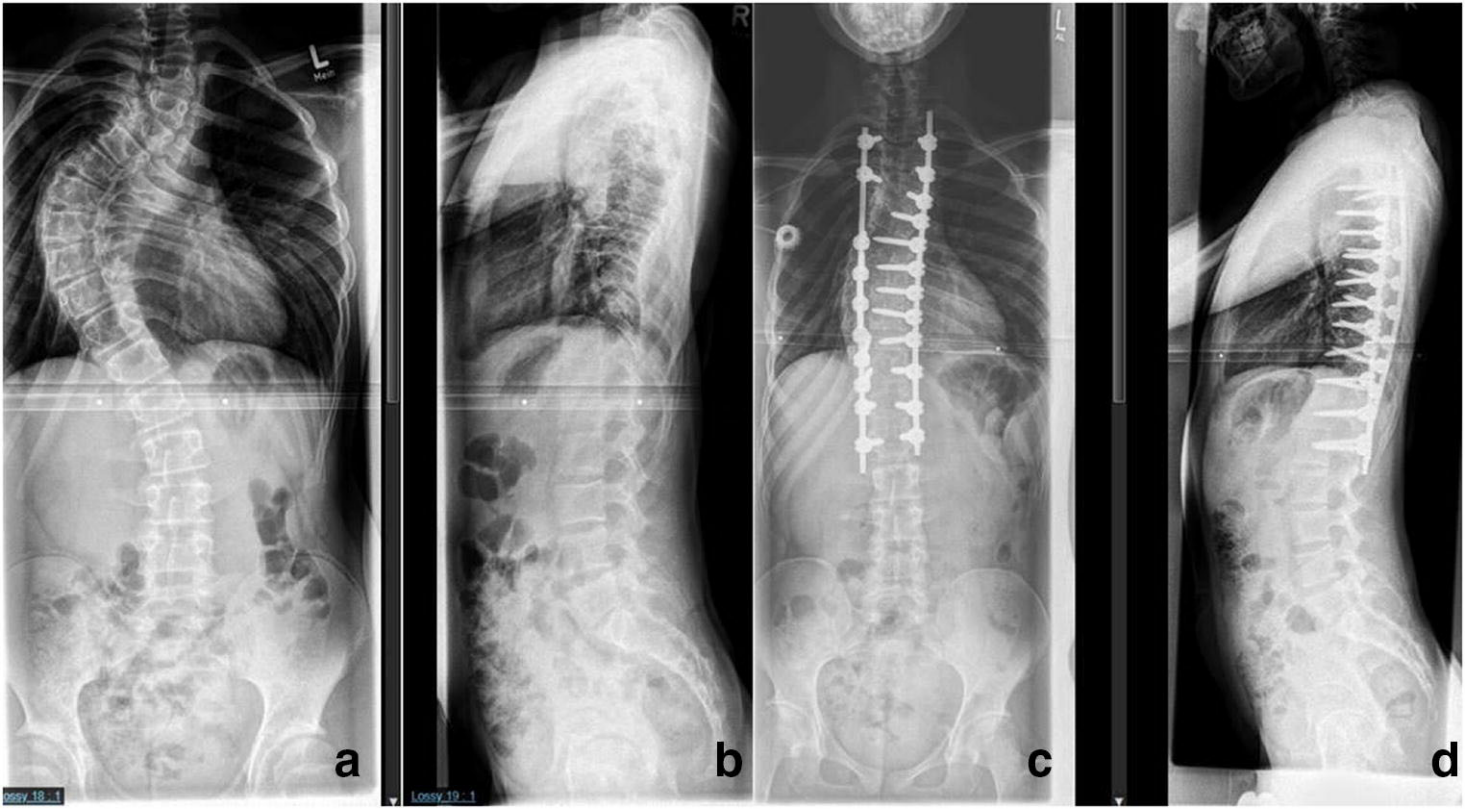
(Case 4) Dystrophic scoliosis of 71° in a 15-year-old patient with NF-1 (**a**, **b**). The curve was flexible on side bending. The patient was treated by posterior spinal fusion T2–L2 with good results on latest follow-up 3 years after initial surgery (**c**, **d**).

Vertebra-based rod instrumentation is the method of choice. Fixation is achieved preferably by means of pedicle screws (Fig. 4). In dystrophic regions with pedicle abnormalities, laminar hooks or sublaminar bands can be used and intraoperative navigation may be very helpful in selected cases. Immobilization in a brace after surgery is usually not necessary.

**Fig. 5:**
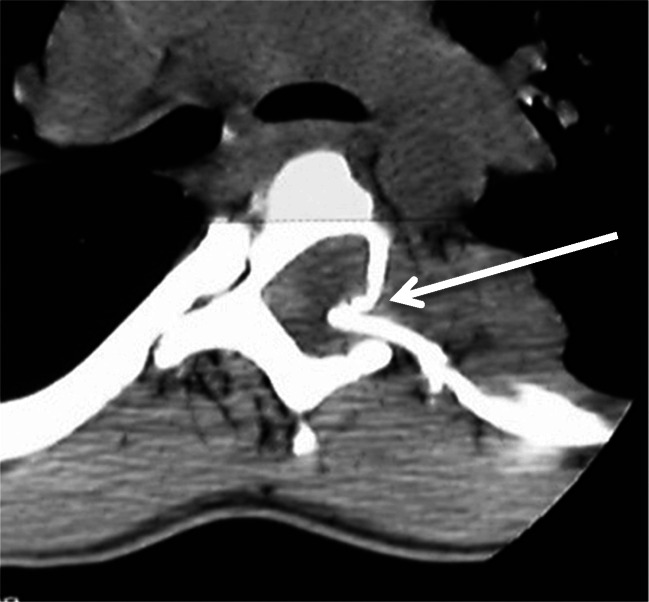
(Case 5) Rib head protrusion into the spinal canal (arrow)

If the curve exceeds 60°, a combined anterior and posterior approach is recommended. The procedure is performed in one surgical setting and good results have been reported [6, 15–18]. However, due to the severe, rigid deformity and poor bone quality, surgery can be very challenging. Meticulous preoperative evaluation and planning is mandatory. The contents of the spinal canal should be evaluated by means of whole spine MRI in order to define critical regions with space-occupying lesions, dural ectasia, or bony erosion of the spinal canal. The vertebral morphology and the evaluation of the possibilities for fixation to the bone are preferably studied with thin-slice CT reconstruction. Special attention is directed to the apical region where dysplastic changes are most pronounced. Careful analysis of the deformity, especially in cases of severe rotational deformities, is mandatory. In these cases, rib head protrusion into the spinal canal through a neuroforamen is a frequent finding and puts the spinal cord at high risk of neurological damage if overlooked (Fig. 5).

In addition, the patient is evaluated for the presence of tumor masses along the planned surgical approach, which may interfere with the exposure of spinal structures. In such cases, interdisciplinary teamwork is of utmost importance.

**Fig. 6:**
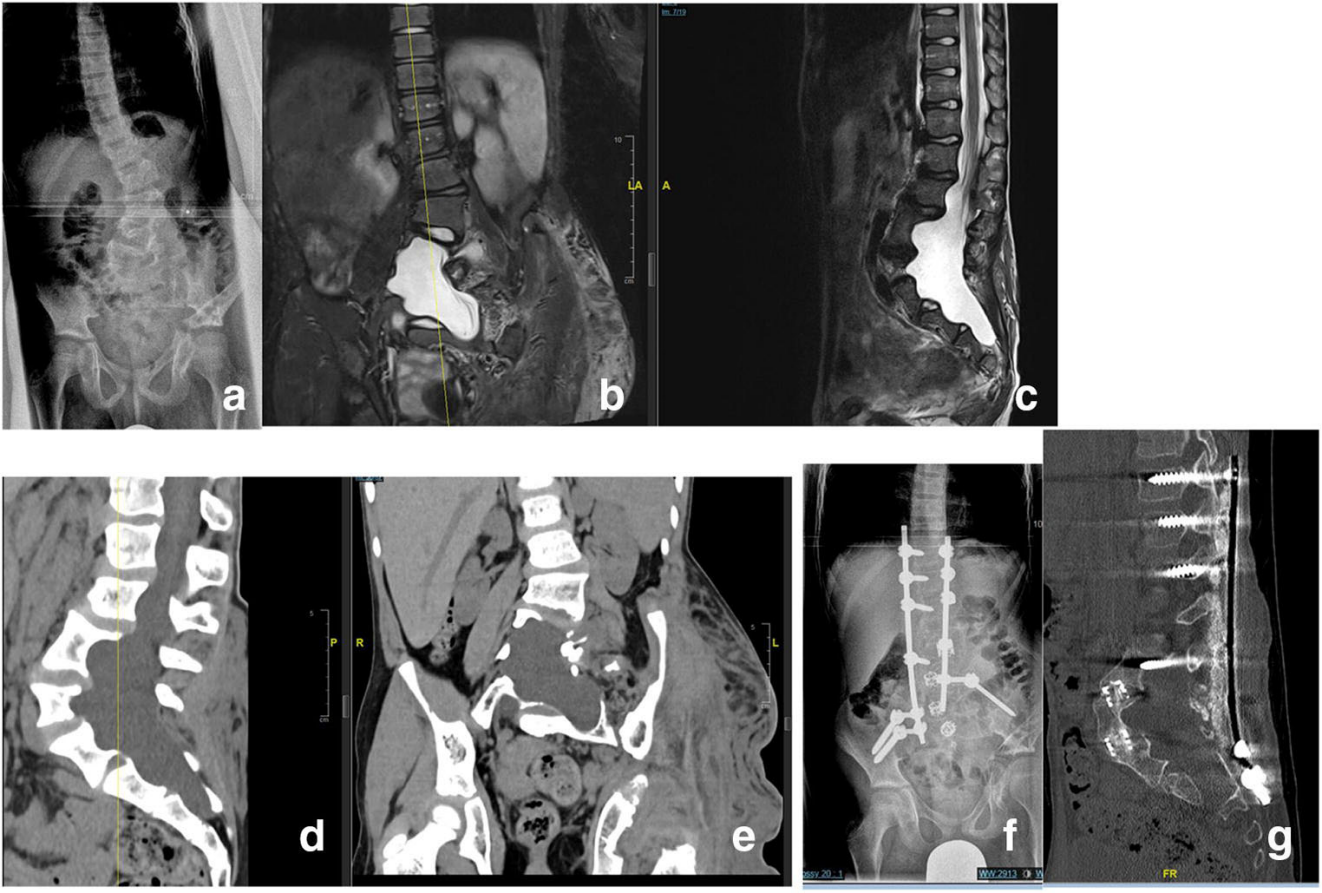
Dystrophic lumbar scoliosis (**a**–**g**) in a 13-year-old NF-1 patient (**a**). Notice the severe dural ectasia visible on MRI (**b**, **c**) as well as the severe bone erosion resulting in a poor bone stock visible on the preoperative CT scan (**d**, **e**).Plain X-ray and noncontrast CT scan after combined anterior and posterior spinal fusion with anterior mesh cages and “off-label” rhBMP-2 showing good correction and stable fusion on latest follow-up (**f**, **g**)

When a combined anterior-posterior approach is indicated critical evaluation of the stability of the vertebral column is of utmost importance. Starting with the anterior part of the procedure has the benefit of achieving more correction through release of the anterior structures. An important prerequisite is a stable spine. If the deformity is potentially unstable, performing the anterior approach first carries the risk of producing additional instability and endangering the spinal cord. If there is a proven or questionable instability, then the posterior approach is performed first in order to stabilize the spine and prevent neurological injury (Fig. 6).

**Fig. 7:**
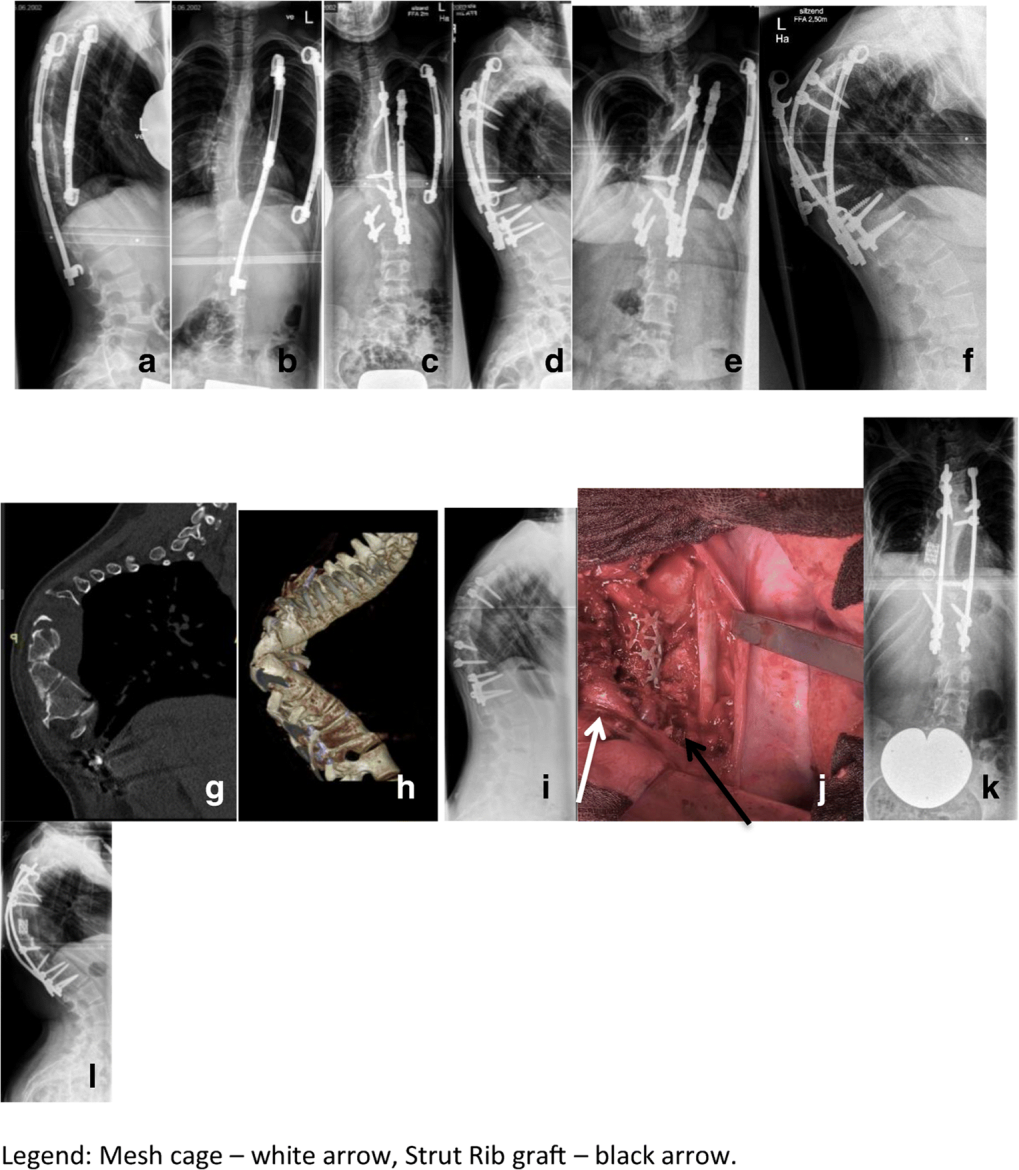
(Case 7) Eleven-year-old NF-1 patient with moderate dystrophic scoliosis (**a**–**l**) . The index procedure comprised a growth-preserving technique by means of vertical expandable titanium ribs (VEPTR). The curve was well-controlled and worsening was prevented for 4 years after initial surgery (**a**, **b**). During the pubertal growth spurt, significant curve progression occurred. An “in situ” posterior fusion combined with concave instrumentation was performed at age 13 (**c**, **d**). On further follow-up, the curve increased significantly due to the “modulation” process of dystrophic alterations (**e**, **f**). Note the severe dystrophic alterations and the complexity of the deformity seen on reconstruction CT scans (**g**, **h**). The patient developed an incomplete flaccid paraplegia of the lower extremities due to an increase of thoracic kyphosis resulting in anterior compression of the spinal cord. The treatment approach comprised of removal of instrumentation and halo-gravity traction for 4 weeks (h1) followed by posterior and anterior spinal fusion. A photo of the anterior procedure showing the intervertebral cages and the strut rib graft in place (**j**). Postoperative X-rays showing good deformity correction (**k**, **l**)

Depending on the levels to be addressed, we perform the anterior part of the procedure through an open transthoracic, a retroperitoneal, or a combined approach from the convex side. The entire dystrophic portion of the spine should be addressed. After exposure of the intervertebral discs, they are meticulously removed. Care should be taken to expose only the subchondral bone but not to violate the endplates since the endplate is the strongest portion of the vertebral body and its damage carries a high risk for loss of anterior column load support. Furthermore, unnecessary exposure of cancellous bone surfaces may result in severe, uncontrolled bleeding. Good anterior support is essential in order to maintain stability. This may be achieved by the use of intervertebral cages if the subchondral bone is intact or by means of autologous cortical strut graft if multiple segments are affected by severe dystrophic alterations and more than 3 segments need to be “bypassed”. If strut grafting is indicated, our preferred technique is a non-vascularized autologous rib or fibula graft, which should be placed into the vertical weight-bearing axis of the spine. The anchor points of the recipient bone should be well-exposed down to bleeding cancellous bone in order to assure adequate blood supply and to promote union. Multiple struts should be placed if possible (Fig. 7).

When performing the posterior approach, care should be taken to expose and decorticate as much as possible from the posterior bony surfaces as a prerequisite for good union. Preparation should be meticulous and all soft tissues should be removed in order to assure good bone graft-recipient contact and to prevent soft tissue interposition between the posterior bony elements and the bone graft. Inadvertent penetration into the spinal canal should be avoided at any price and exposure is performed cautiously and preferably by electrocautery in order to control bleeding and in order to avoid violating the dystrophic bone during subperiosteal preparation, especially if bone erosion is present and significant dural ectasia is seen on preoperative MRI [19–21].

Posterior fusion should extend from the neutral cranial to the neutral caudal vertebra. Vertebra-based dual rod segmental instrumentation is the method of choice and pedicle screw anchors are preferred since these provide the most stable fixation. In the presence of bone erosion and pedicle dystrophy, hooks or sublaminar bands may be used, but care should be taken not to violate the dura. On occasion, segments affected by severe dystrophy may be spared from placing anchors and a “bypassing/bridging” instrumentation technique can be performed. Abundant autologous iliac crest cancellous bone graft is applied. The use of biologic osteoinductive substances such as rhBMP-2 is implemented on an individual basis. We use rhBMP-2 during the index procedure after critical evaluation of the probability to obtain fusion in the first 6 months after surgery. The most important risk factors for the anticipation of delayed union is the presence of severe dystrophic changes with bone erosion resulting in an insufficient residual contact bone surface. Our empiric criteria for contact insufficiency are anterior intervertebral contact of less than 1cm^2^ and/or a bone gap between the posterior elements of more than 1 segment.

A brace is applied for 6 months after surgery in an effort to promote stability and reduce the risk of pseudarthrosis.

In the presence of severe dystrophic alterations the fusion mass should be routinely evaluated by a CT scan 6 months after index surgery. In case of delayed union, fusion mass augmentation with generous autologous cancellous bone should be performed. Additionally, the application of BMP should be discussed.

Human recombinant BMP-2 was used in 8 patients in our series. No complications related to BMP application were observed and all patients went on to develop solid fusion. However, due to the small patient number we do not have representative statistical data to support an increase in fusion rates that can only be attributed to the use of BMP. BMP use in the pediatric population remains “off-label” and is decided at the discretion of the surgeon. Its use should be avoided or discussed individually in case of existing tumor masses due to the risk of malignant transformation of neurofibromas.

A pseudarthrosis rate after attempted spinal fusion of up to 31% has been reported in NF-1 patients [22].

In our series, one patient with cervical kyphosis and instability due to severe dystrophic changes showed delayed union and needed augmentation of the fusion mass. Solid fusion occurred at the latest follow-up.

Excessive bleeding has to be anticipated in NF-1 patients undergoing spinal surgery. This is due to the underlying bone dystrophy and the high vascularity of the soft tissue tumors. Besides meticulous hemostasis we routinely implement a cell saver for autologous blood transfusion if there are no signs of malignant transformation in preoperative imaging. Enough blood products for homologous transfusion should always be available. In our series, all patients receiving fusion required blood transfusions.

Higher rates of postoperative neurological deficits have been reported in NF-1 patients, especially in cases with kyphosis. Because of the higher risk of paraplegia in these patients, some surgeons tend to perform laminectomy in order to decompress the dura. We cannot support this approach because posterior decompression will not resolve the anterior compression of the spinal cord, which is always present in cases with kyphotic deformity. In our opinion laminectomy is contraindicated since it does not resolve the problem of anterior compression and because removal of posterior bony structures destabilizes the spine additionally with the added potential risk of an increasing deformity and it increases the risk of neurological damage. It needs to be emphasized that laminectomy has been proven to be ineffective to reduce compression of the spinal cord in angulated kyphotic deformities [8].

In order to reduce the risk of neurologic deficit, we recommend the following:

- Preoperative MRI imaging of neural structures (whole spine MRI) to exclude intraspinal pathologies
- Neurosurgical removal of intraspinal tumors before treatment of the spinal deformity, if indicated
- Gradual correction of severe deformity by means of halo-gravity traction
- Routine use of intraoperative neuro-monitoring included TcMEP
- Maintenance of spinal cord perfusion by means of adequate perioperative blood pressure and Hb level management

The use of preoperative halo-gravity traction (HGT) for the treatment of dystrophic spinal deformity due to NF-1 needs special attention. The rationale of preoperative HGT is a continuous longitudinal distraction in order to achieve slow deformity correction and to avoid abrupt distension of the neuro-vascular structures and thus minimize the risk of neurologic compromise. In addition, since a significant amount of the deformity is gradually corrected prior to the surgical procedure, there is no need to apply strong corrective forces on the bony anchors of the spinal instrumentation especially in the presence of dystrophic bone changes. Using this strategy, the risk of mechanical complications such as implant pull out are significantly reduced. The patient is admitted to the hospital for halo application 4 weeks before scheduled spinal surgery. Continuous traction (24/24 h) is started the same day after halo application with 10% body weight (BW) and is increased daily by additional 5–10% BW up to 60% BW. Traction is exercised in both the supine and upright positions. The patient is supplied with an individually fitted wheelchair and with a posterior walker, which allows for continuous traction in the standing and sitting positions. Neurological examination including cranial nerve function is monitored every 8 h. X-ray controls under traction are performed once a week in order to monitor the progress of deformity correction.

Benefits of slow correction of the deformity through preoperative halo-gravity traction are:

- Preservation of spinal cord perfusion through avoidance of acute stretching of the neural structures and blood vessels
- Application of strong corrective forces during surgery can be avoided since most of the deformity is already corrected by preoperative traction. This is very important especially in cases with severe dystrophic alterations and poor bone stock.

Our indications for HGT are if at least one of the following criteria is present:

- Severe scoliosis of > 90°
- Rigid scoliotic curve which does not straighten to under 50° on side bending test
- Kyphosis of > 45° in the C-spine or > 90° in the thoracic spine

Key points for the treatment of spinal deformities:

- Careful evaluation for radiographic dystrophic signs is of utmost importance
- Dystrophic curves will progress and require surgical treatment
- Growth-preserving instrumentation combined with short segment apical fusion is the treatment of choice in the immature patient
- Halo-gravity traction is very efficient for gradual correction of severe rigid curves
- skeletally mature patients with severe dystrophic curves exceeding 60° need anterior and posterior spinal fusion, especially if kyphotic deformity is present
- Higher rate of peri- and postoperative complications such as bleeding and delayed union is to be expected in patients with spinal deformity and NF-1.

## Tibial dysplasia in NF-1 patients

### Incidence

Tibial dysplasia has been observed in 5% of NF-1 patients and represents the second most common osseous manifestation after spinal deformities. It has been reported that 84% of the patients presenting with congenital pseudarthrosis of the tibia (CPT) match the criteria for NF-1 [18, 23].

### Etiology

The exact reason for the involvement of the tibia is still unknown. Histologic studies found that pseudarthrosis was surrounded by a thick cuff of hamartomatous tissue. Hypotheses suggest that cells from this hamartomatous tissue do not undergo osteoblastic differentiation and show high osteoclastic activity [24, 25].

Genetic studies identified 80% of the patients with CPT as carrying pathogenic variants of the NF-1 gene. A higher proportion of these “de novo” mutation carriers presented bone fractures as compared to inherited variant carriers. The double inactivation of the NF-1 gene with subsequent clonal growth was suggested as a possible pathophysiologic mechanism at least in some of the CPT patients. However, these data need still to be confirmed in larger studies [26, 27].

### Clinical picture

Clinical presentation is often in early childhood. In cases of simple tibial dysplasia, an anterior or antero-medial bowing of the tibia with some degree of limb shortening is present. In the case of fracture or frank pseudarthrosis, there is pathological movement between the fragments, which is usually not as painful as could be expected in a normal fracture. The plain X-ray study is the imaging method of choice for initial evaluation and should be performed in any child with NF-1 presenting with non-physiologic bowing of the leg. Radiological appearance varies from simple anterior tibial bowing accompanied from dystrophic changes of bone up to a complete pathological fracture. The pseudarthrosis may be present at birth or may occur with time due to the progressive bone dystrophy.

MRI is very helpful to define the extent of bone involvement and is mandatory for preoperative evaluation, especially if surgical resection and reconstruction is planned.

### Management

The main issues encountered in the management of CPT are of mechanical and biological origin: the deformity is severe, fixation and stabilization with implants is difficult due to poor bone stock and dystrophic changes, and the biological properties for new bone formation and healing are poor.

Management of tibial dysplasia without fracture aims at preventing fracture. Bracing of young children is currently the gold standard and should not be discontinued until skeletal maturity [28].

However, fracture prevention in all cases with bracing alone remains elusive. In order to reduce the risk of fracture, a prophylactic “bypass grafting” has been suggested [29]. In those cases an allograft fibula was inserted during surgery on the concave side of the bowing along the weight-bearing axis. The presumption is to “bypass” the dystrophic portion of the bone in order to stabilize the tibia. With this approach fracture prevention was achieved in 70 to 100% of the reported cases; however, patient cohorts were anecdotally small (a total of 19 patients) [30, 31].

Treatment strategies for established fractures with pseudarthrosis aim to obtain solid union, prevent repeat fracture, correct axial deformity, and equalize limb lengths. Initial results with surgical treatment of CPT were very discouraging. Union was obtained in only 1.5% of cases and almost one-third of the patients required amputation [32].

Modalities of management of CPT evolved over the past decades and current treatment protocols comprise excision of the pseudarthrosis and the surrounding hamartoma, autogenous bone grafting, and adequate intramedullary fixation. There is a consensus that pathologic bone and hamartoma tissue as well as the thickened periosteum at the pseudarthrosis site should be completely excised. Different techniques have been described for reconstruction of the osseous defect, including vascularized or non-vascularized autologous bone grafts (usually the contralateral fibula). Another option is bone segment transport based on the principles of distraction osteogenesis. Similar results with all three techniques have been reported; however, there is currently no strong evidence in favor of one of the methods due to the small number of cases [33–35].

Stable fixation is essential after resection of the pseudarthrosis. Plating, intramedullary nailing, external fixation, or a combination of the above methods have been used. Intramedullary nailing fixation provides good initial fixation. It is recommended that the nail be left in place until skeletal maturity in order to prevent fracture. Since the bone will grow with time, telescopic intramedullary nailing is currently the preferred method of choice in an immature individual as longitudinal bone growth continues.

It should be emphasized that external fixation is a very useful technique in the treatment of CPT as an additional option for temporary stabilization. With this method, both fragments can be aligned and held under compression in order to promote bone healing and achieve union. If needed, a simultaneous leg lengthening or a segment bone transport as well as concurrent angular correction of an axial deformity can be performed. Currently, external ring fixators are routinely used in combination with intramedullary nailing. After solid union is achieved the external fixator is removed and the intramedullary nail is retained for the purpose of fracture prevention. If the distal bone fragment is too short a temporary crossing of the ankle joint with the nail can be performed. However, this should be avoided if possible since this may produce ankle stiffness and muscular weakness [36].

Bone grafting is essential for successful union. However, there is still no consensus about the type of bone graft to be used. Cancellous bone has better osteogenic potential but is much more susceptible to resorption. On the other hand, cortical strut grafts have better resistance to resorption and acceptable osteogenic potential especially if used as a vascularized graft. Drawbacks include the associated morbidity on the harvesting site (usually the healthy extremity), lengthy surgery, and the challenging microsurgical technique. Due to the small size of the structures, internal fixation may be extremely difficult if a cortical graft is used, especially in a small child. Synthetic osteoinductive substances such as recombinant human bone morphogenic proteins (rhBMP-2, rhBMP-7) are being increasingly implemented to enhance bone formation. However, their use in the pediatric population is currently “off-label”. The decision for BMP use is made on an individual basis at the discretion of the surgeon since evidence is not available and there are concerns about a theoretical risk of malignancy [37, 38].

The appropriate age to perform surgery is still under discussion. In a large European multicenter study including 340 patients from 13 countries, it was strongly recommended to avoid surgery under 3 years of age and even to postpone surgery if possible until the age of 5 years [39].

However, it should be emphasized that the poor results in this study may be biased by the surgical methods since most of the patients were immobilized after resection of the pseudarthrosis only by means of an external fixator and had no intramedullary stabilization. In contrast to these observations, very good results were most recently reported if initial surgery was done before the age of 5 years [40].

Overall good functional results were reported in approximately 80% of the cases if the treatment approach comprised a combination of pseudarthrosis resection, autogenous bone grafting, and intramedullary nailing.

The risk of re-fracture after initially obtained union should not be underestimated since re-fracture was observed in up to 50% of the patients. According to the current literature half of these re-fractures healed with cast treatment only and the other half needed repeat surgery [41, 42].

Primary amputation for CPT is not justifiable since good results have been reported with reconstructive limb preserving methods. Amputation should be performed only in exceptionally rare cases when multiple reconstructive attempts failed to achieve a functionally stable union.

Our treatment strategy in tibial dysplasia and CPT due to NF-1 is as follows:

- Tibial dysplasia with bowing, without fracture:

Bracing in order to prevent fracture. A well-fitted “double shell” custom-made brace is prescribed, which should be worn in the upright position until skeletal maturity. If the dystrophic changes and the bowing comprise the lower third of the tibia, the ankle joint and the foot are included in the brace. Clinical and X-ray controls are performed on a yearly basis.

- Tibial dysplasia with fracture:

If the fracture occurs before the patient can walk, surgery is delayed until the child is 18 months old. A custom -made brace is provided for support. MRI is performed at age 1 year in order to define the dystrophic segment of bone to be resected.

If the child is older than 18 months at initial presentation the surgery can be scheduled without delay.

The surgical procedure includes:

- Complete surgical resection of the hamartoma tissue and dystrophic bone segment
- Intramedullary fixation with a telescoping nail
- Crossing of the ankle joint is to be avoided if possible
- External ring-fixation for additional stability
- Distraction osteogenesis for bone segment transport in order to fill the gap of resected bone and preserve leg length
- Docking procedure after fragment contact is achieved, combined with abundant autologous cancellous iliac crest bone graft and optional rhBMP-2 application
- After solid union is obtained, the external fixator is removed and the intramedullary rod is retained.

A brace is used until skeletal maturity. Clinical and X-ray controls are performed on a yearly basis.

We present our single institutional experience of 14 patients with tibial dysplasia associated with NF-1.

Patient demographics are summarized in Table 3.

**Table 3:** Patient demographics for tibia pseudarthrosis

No.	Age initial	Presentation	History	Management	Time to union	No. of surgeries	Re-fracture	Follow-up years
1	5 years	CPT		PR, ERF	6 months	1		12
2	21 years	CPT	Multiple previous surgeries	PR, ERF, BMP	5 months	n.n		1
3	5 years	Bowing	Fracture	OT, IMN, BMP		1		7
4	1 years	CPT		PR, ERF, IMN, BMP	1 year	4	1	17
5	10 years	Bowing	Fracture	Cast, Brace	2.5 years			3
6	2.5 years	Bowing	Stable	Brace				4
7	1 years	CPT		Brace, Surgery pending				< 1
8	3 years	Bowing	Stable	Brace				1
9	1 years	Bowing	Stable	Brace				3
10	3 years	Bowing	Stable	Brace				2
11	2 years	Bowing	Fracture despite”bypass” surgery	By-pass, PR, ERF, BMP	9 months	3		4
12	2 years	Bowing	Fracture	PR, ERF, IMN, BMP	7 months	3×		2
13	3 months	CPT		Brace, Surgery pending				< 1
14	5 years	Bowing	Stable	Brace				2

*PR* pseudarthrosis resection, *RFE* external ring fixator, *IMN* intramedullary nail, *BMP* bone morphogenic protein

n.n the patient had multiple previous surgeries but the exact number of the surgeries was not known

Five of the patients (*n* = 5) presented initially with tibia pseudarthrosis due to pathological fracture. The remaining 9 cases showed anterior tibial bowing of different degrees. In five of them (*n* = 5), bowing remained stable over time and 4 progressed to a pathological fracture despite bracing. In one patient the fracture healed with casting and successive bracing. Six patients received surgical treatment, and in 2 other patients, the surgery is pending. Surgical procedures consisted of resection of the pseudarthrosis and autologous bone grafting combined with rhBMP-2 application (*n* = 5), intramedullary nailing (*n* = 4), and application of a ring fixator (*n* = 4). In all patients, solid union was achieved. Time to achieve union varied between 5 and 13 months. Four patients (*n* = 4) needed more than one surgery in order to achieve fusion. Re-fracture occurred in 1 patient and was treated surgically. All surgically treated patients showed a good functional result at latest follow-up with full weight bearing in a brace and no restriction of daily living (excluding competitive sports) at latest follow-up.

### Conclusion

**Fig. 8:**
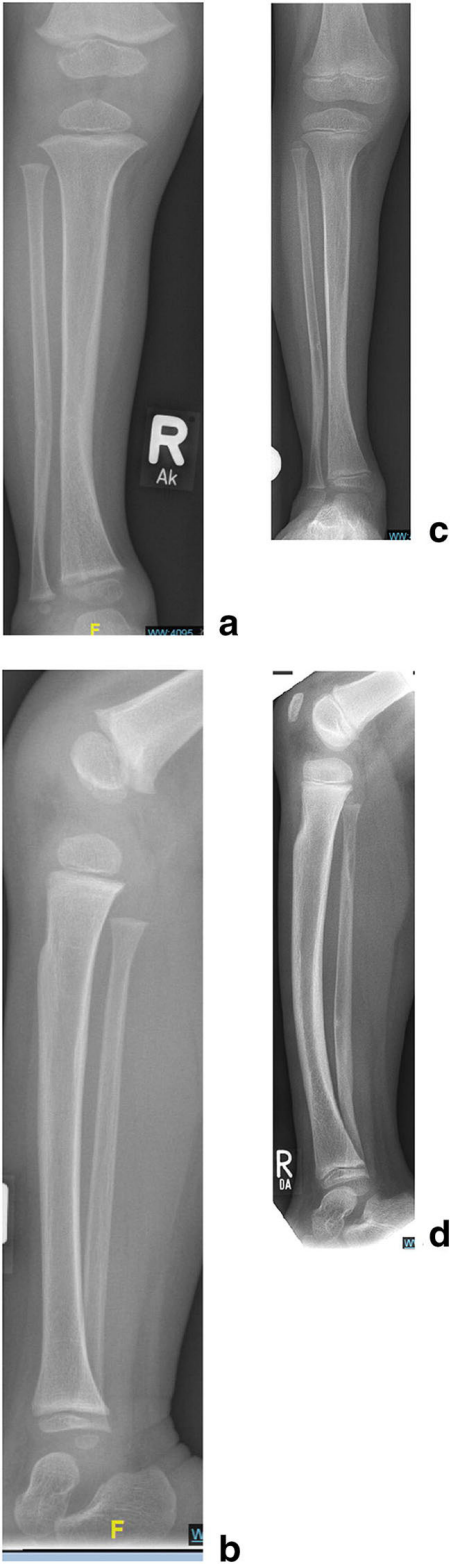
(Case 8) Tibial dysplasia (**a**–**d**). Tibial bowing in a 2-year-old child with tibial dysplasia (**a**, **b**). Brace management was prescribed. Follow-up after 3 years. The lateral view shows minor progression of anterior bowing but no significant worsening of the dysplastic changes with good intramedullary-cortical differentiation (**c**, **d**). Further brace wear was recommended

**Fig. 9:**
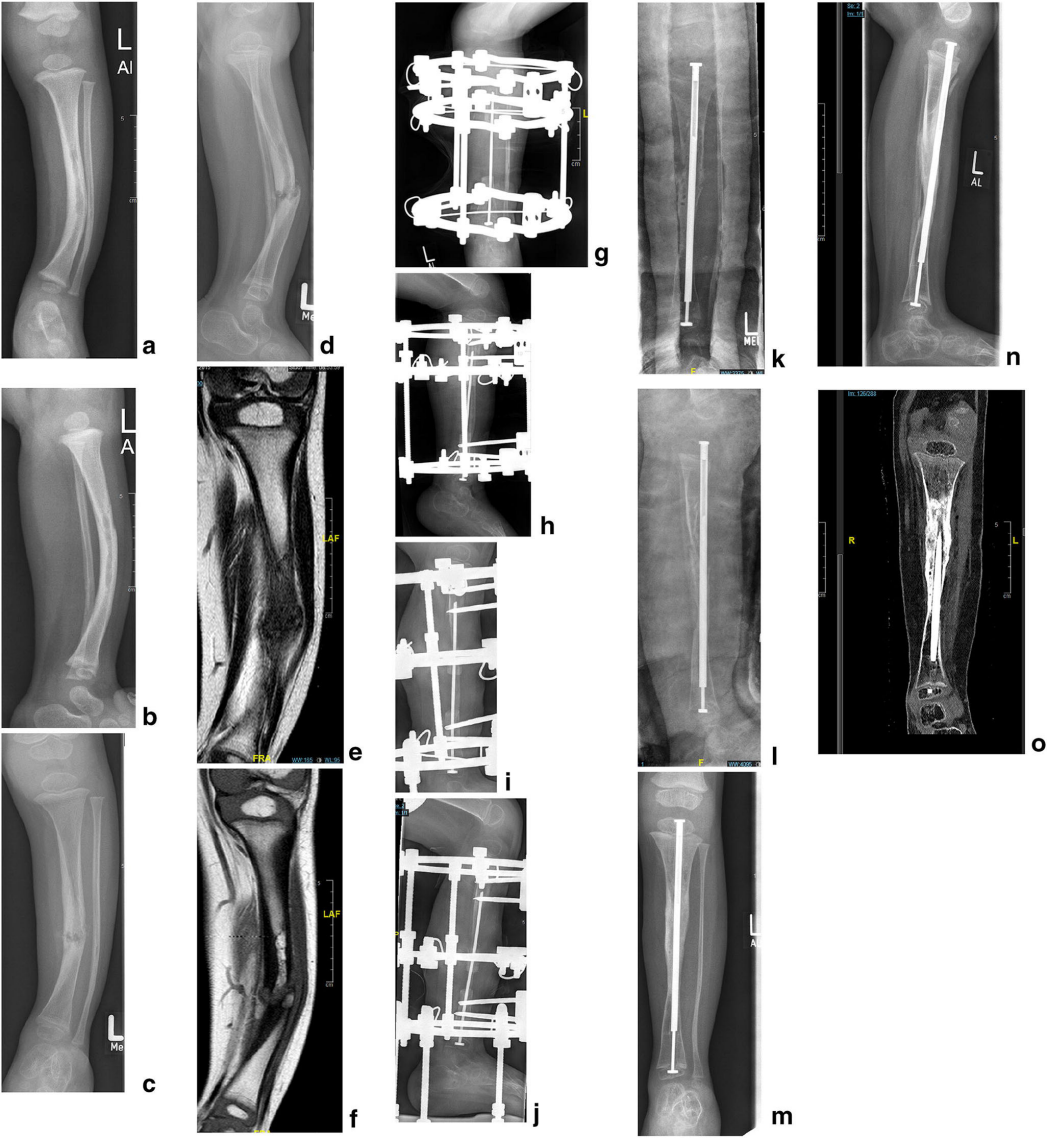
(Case 9) Antero-lateral tibial bowing (**a**–**o**) in a 3-year-old child (**a**, **b**). Pathological fracture at age 4.5 years despite bracing (**c**, **d**). The extent of dystrophic changes is seen on MRI (**e**, **f**). Resection of the pseudarthrosis, retrograde intramedullary nailing and application of a ring fixator for segment bone transport in order to fill the defect (**g**, **h**). Bone segment transport with concurrent distraction osteogenesis(**i**, **j**). Six weeks after the “Docking procedure” after completion of the bone segment transport with telescopic intramedullary nail osteosynthesis and rhBMP-2 application. The external ring fixator is already removed (**k**, **l**). Final result with solid union a.p. (**m**), lateral (**n**), CT scan (**o**). The intramedullary telescopic nail is retained, the child should wear a protective “double shell” brace until maturity.

**Fig. 10:**
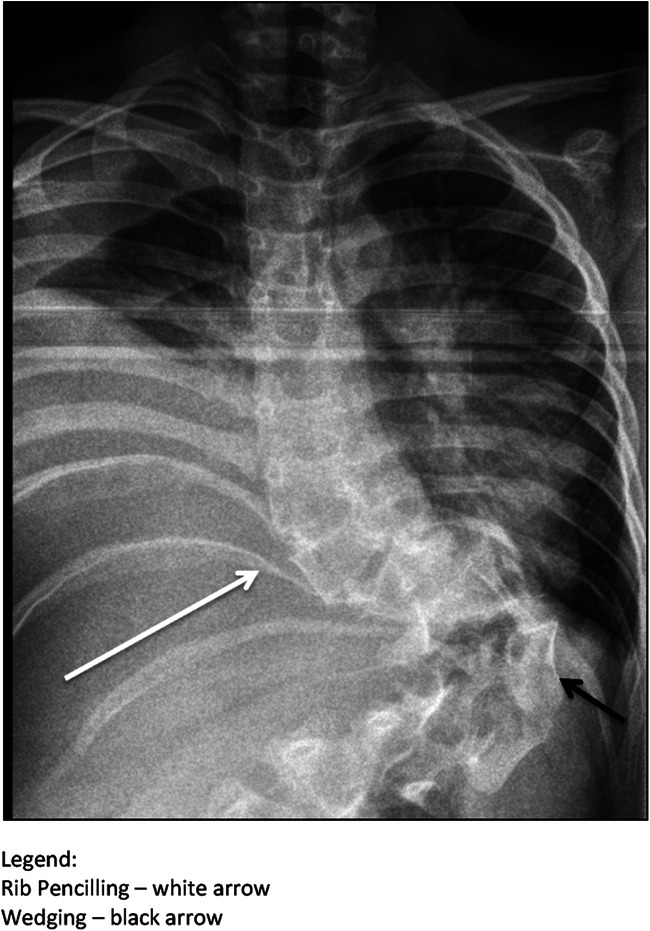
Typical appearance of a short-segment dystrophic scoliosis in a patient with NF-1

**Fig. 11:**
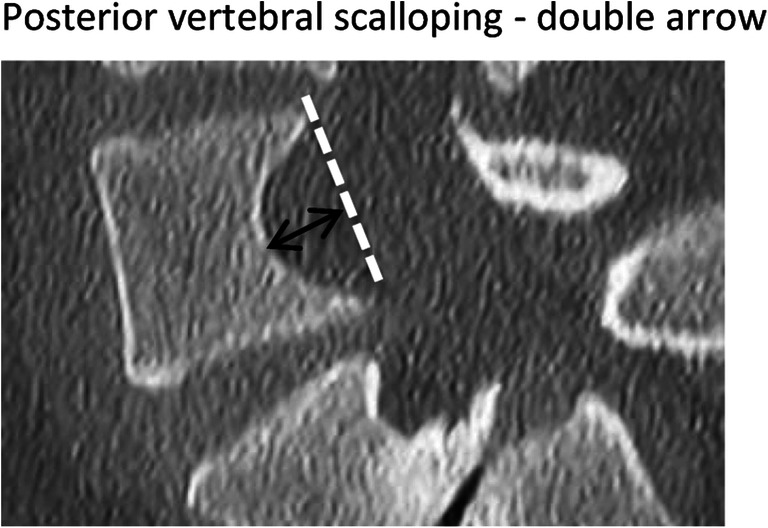
Typical appearance of posterior vertebral scalopping

Based on the current knowledge on CPT management in NF-1 patients it can be stated that fracture union with satisfactory functional results can be obtained in over 80% of cases. The recommended surgical protocol consists of resection of the pseudarthrosis, intramedullary nailing, and bone grafting. However, there is no evidence to support this statement since level 1 studies are not available. “Off-label” BMP application remains optional. Brace wear until skeletal maturity is mandatory in order to minimize the risk of re-fracture (Figs. 8, 9, 10, 11).
